# The complete chloroplast genome sequence of *Lophatherum gracile*

**DOI:** 10.1080/23802359.2020.1839365

**Published:** 2020-12-24

**Authors:** Liqiong Jiang, Hua Peng, Yuehua Wang

**Affiliations:** a School of Ecology and Environmental Sciences and School of Life Sciences, Yunnan University, Kunming, China; b Yunnan Key Laboratory of Plant Reproductive Adaptation and Evolutionary Ecology, Yunnan University, Kunming, China; c CAS Key Laboratory for Plant Diversity and Biogeography of East Asia, Kunming Institute of Botany, Chinese Academy of Sciences, Kunming, China

**Keywords:** *Lophatherum gracile*, chloroplast, Illumina sequencing, phylogenetic analysis

## Abstract

The first complete chloroplast genome (cpDNA) sequence of *Lophatherum gracile* Brongn. was determined from Illumina HiSeq pair-end sequencing data in this study. The cpDNA is 140,595 bp in length, contains a large single-copy region (LSC) of 82,447 bp, and a small single-copy region (SSC) of 12,626 bp, which were separated by a pair of inverted repeats (IR) regions of 22,761 bp. The genome contains 130 genes, including 83 protein-coding genes, eight ribosomal RNA genes, and 39 transfer RNA genes. The further phylogenomic analysis showed that *L. gracile* and *Zeugites pittieri* clustered in a clade in Poaceae family.

The genus *Lophatherum* Brongn. comprising two species, of which *L. gracile* Brongn. is widely distributed in warm-temperate and tropical Asia. This species is a perennial herb with spindle-shaped root tubers distribute in China, Cambodia, India, Indonesia, Japan, Korea, Malaysia, Myanmar, Nepal, New Guinea, Philippines, Sri Lanka, Thailand, Vietnam, Australia and Pacific Islands (Liu and Phillips 2006). *Lophatherum gracile*, an important medicinal plant, is used traditionally in the treatment of cough associated with lung heat and inflammation (Zhang et al. 2010; Shao et al. 2014; Chen et al. 2019). However, there has been no genomic studies on *L. gracile*.

Herein, we reported and characterized the complete *L. gracile* plastid genome. The GenBank accession number is MT876425. Fresh leaves of *L. gracile* were collected from Tianbao Town, Malipo County, Yunnan, China (104.724035°E, 23.039173°N). Voucher specimen (Jiang & Chen. jlq140) was kept in the Herbarium of Kunming Institute of Botany, Chinese Academy of Sciences (KUN) and the accession number is 1345291. DNA was extracted from its fresh leaves using DNA Plantzol Reagent (Invitrogen, Carlsbad, CA, USA).

Paired-end reads were sequenced by using Illumina HiSeq system (Illumina, San Diego, CA). In total, about 22.1 million high-quality clean reads were generated with adaptors trimmed. Aligning, assembly, and annotation were conducted by CLC de novo assembler (CLC Bio, Aarhus, Denmark), BLAST, GeSeq (Tillich et al. 2017), and GENEIOUS v11.0.3 (Kearse et al. 2012). To confirm the phylogenetic position of *L. gracile*, the other 19 species of Poaceae family from NCBI were aligned using MAFFT v.7 (Katoh and Standley 2013). The Auto algorithm in the MAFFT alignment software was used to align the 20 complete genome sequences and the G-INS-i algorithm was used to align the partial complex sequences. The maximum-likelihood (ML) bootstrap analysis was conducted using RAxML (Stamatakis 2006); bootstrap probability values were calculated from 1000 replicates. *Eragrostis tenellula* (NC042833), *Isachne albens* (MF035986), *Arundo donax* (NC037077) and *Phragmites australis* (KJ825856) served as the out-group.

The complete *L. gracile* plastid genome is a circular DNA molecule with a length of 140,595 bp, contains a large single-copy region (LSC) of 82,447 bp and a small single-copy region (SSC) of 12,626 bp, which were separated by a pair of inverted repeats (IR) regions of 22,761 bp. The overall GC content of the whole genome is 38.6%, and the corresponding values of the LSC, SSC, and IR regions are 36.5%, 32.8%, and 44.1%, respectively. The plastid genome contained 130 genes, including 83 protein-coding genes, eight ribosomal RNA genes, and 39 transfer RNA genes.

The further phylogenomic analysis showed that *L. gracile* and *Zeugites pittieri* clustered in a clade in Poaceae family (Figure 1). The determination of the complete plastid genome sequences provided new molecular data to illuminate the tribe Zeugiteae evolution.

**Figure 1.**
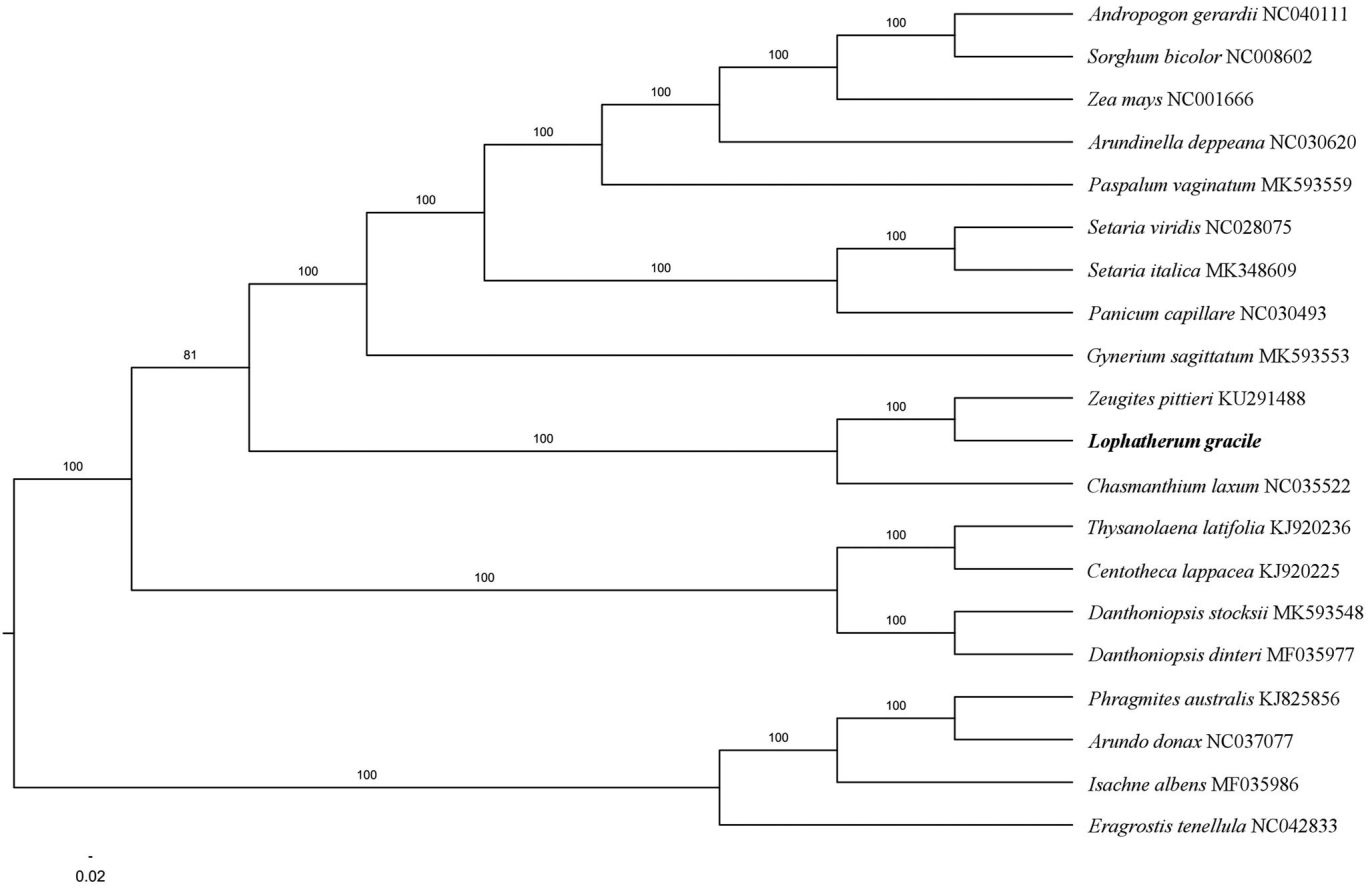
The maximum-likelihood tree based on the 20 chloroplast genomes of Poaceae. The bootstrap value based on 1000 replicates is shown on each node.

## Data Availability

The data that support the findings of this study are openly available in the NCBI GenBank database (https://www.ncbi.nlm.nih.gov), and the accession number of the raw sequencing reads is SRR12616405. Unrestricted use, distribution, and reproduction of the data is permitted in any medium, provided the original work is properly cited.
